# Porosity and dielectric properties as tools to predict drug release trends from hydrogels

**DOI:** 10.1186/2193-1801-3-393

**Published:** 2014-07-29

**Authors:** Iruthayapandi Selestin Raja, Nishter Nishad Fathima

**Affiliations:** Chemical Laboratory, Central Leather Research Institute, Council of Scientific and Industrial Research, 600020 Adyar, Chennai, India

**Keywords:** Porosity, Impedance, Gelatin-chitosan hydrogels, Catechin, *In vitro* drug delivery

## Abstract

Conventional studies on hydrogel properties such as viscosity, pH and swelling provide information without treating the components of hydrogel, viz., water and polymer individually. Water and hydrophilic polymers need to be studied individually to understand their relationship with each other to relate their influence on drug release. In this context, we have assigned the combination of porosity and dielectric properties as tools to explore the hydrogels. Porosity and dielectric properties have been analyzed using thermoporometry and alternative current impedance measurements, respectively. A well-known hydrogel genipin cross linked gelatin-chitosan (GC) composite, with catechin as model drug has been studied. The increasing concentration of chitosan in the hydrogel composites led to increase in bound water content and incorporation of charge entrapping moieties. Controlled and medium drug release are observed for GC1 whereas the native hydrogels and composites with lower ratio of chitosan yield immediate release and composites with higher ratio effects in slow release for limited duration (9 hours) of drug delivery process. This trend of drug release is in accordance with the results obtained from porosity and dielectric properties where reduction in pore radii to lower range and increase in relaxation time of polymeric components were observed at higher concentration of chitosan. Thus, these properties can be judiciously used for predicting drug release and designing biomaterials according to it.

## Background

Hydrogels consist of hydrophilic polymers dispersed in water medium and hence termed as colloidal solution (Sarkhejiya and Baldaniya 2012). The release of drug molecules from a hydrogel matrix is usually predicted using the properties such as swelling, viscosity, pH, biodegradation and degree of cross linking (Cheng et al. 2014; Soni and Singhai et al. 2013; Sun et al. 2013; Zhang et al. 2013). Though these properties emphasize the whole hydrogel system itself, they fail to characterize the individual components present in it. Porosity and dielectric properties bear advantage over other properties as the impact of water is highlighted for pore size determination and that of polymeric component is stressed for analyzing admittance and dielectric relaxation (Kanungo et al. 2011; 2013a).

Porosity measurement using thermoporometry gains advantage over other techniques such as Brunauer-Emmett-Teller (BET) and Mercury Intrusion Porosimetry (MIP), as the latter demands the sample to be in dried state (Chae et al. 2013; Fathima et al. 2002; Qu et al. 2006). Thermoporometry determines pore size based on melting or crystallization point of water molecules confined into the pores of hydrogels. Analyzing thermodynamic behavior of water with respect to its interaction with polymers helps evaluate different types of water and hence provide a range of pore sizes in nano range (Landry 2005; Yamamoto et al. 2005a, [b]). Alternative Current (AC) impedance analysis is a promising tool to explore dielectric properties of bio materials. Hydrogels are present in human body in the form of mucus, cartilage and vitreous humor of eye (Dean et al. 2008; Schaefer et al. 2002) and are acting as dielectric materials (Ren and Lam 2008).

Gelatin is a well-known protein and is a denatured form of collagen (Zeugolis and Rahunath 2010). As breakdown products of gelatin are not harmful for human beings, they are known to be biocompatible (Pulieri et al. 2008). Gelatin has been widely used for various types of biomedical applications such as contact lenses, wound dressing, food additives including drug delivery (Dongargaonkar et al. 2013; Ngo et al. 2014; Shi and Tan 2004). Chitosan is a linear polysaccharide consisting of reactive amine and hydroxyl groups. Chitosan and its derivative have been proven as safe candidates for mucosal and trans- mucosal delivery of drugs (Cui et al. 2014; Rodrigues et al. 2012). Hence, in this present study, gelatin-chitosan composite hydrogels have been taken as model system. Though composites are preferred in many of the applications to enhance physical properties of native hydrogels in the aspect of elasticity, tensile strength and thermal stability (Parvez et al. 2012; Yin et al. 2010), the rationale behind selecting copolymer, in our work, is to enhance porosity attaining well cross linked network for better drug release profile. For the purpose of bringing about permanent cross linkage between polymers, genipin, a biological cross linking agent is taken during composite preparation. Genipin involves in covalent bond formation between free NH_2_ groups found in lysine and arginine of gelatin and present on each residue of chitosan. The pH value 7 and temperature above 40°C enhance the rate of reaction to form genipin dimer between polymers. The proposed formation structure of blue pigments (genipin dimer) from genipin is shown in previous reports (Chaubaroux et al. 2012; Wang et al. 2012).

Hydrogels are one of the most widely used drug delivery agents for various kinds of drugs such as anti-oxidant, hormone and protein (Park et al. 2013; Peng et al. 2012; Van Thienen 2007). Catechin, a free radical scavenger containing hydroxyl groups is taken as model drug for *in vitro* study. It is reported that catechin accounts for low bioavailability when administered orally. It is metabolized into conjugated mainly methylated form, which is not active against oxidative stress (Baba et al. 2001; Veluri et al. 2004). When the anti-oxidant encapsulated into hydrogels scaffold is injected intravenously, the availability of active ingredients of catechin in blood can be increased.

The objective of this work is to elucidate the dispersion medium (water) and dispersed phase (polymers) of hydrogels through porosity and dielectric properties and to relate the effect of these properties with drug release profile.

## Results and discussion

### Porosity and dielectric properties of hydrogels

**Figure 1:**
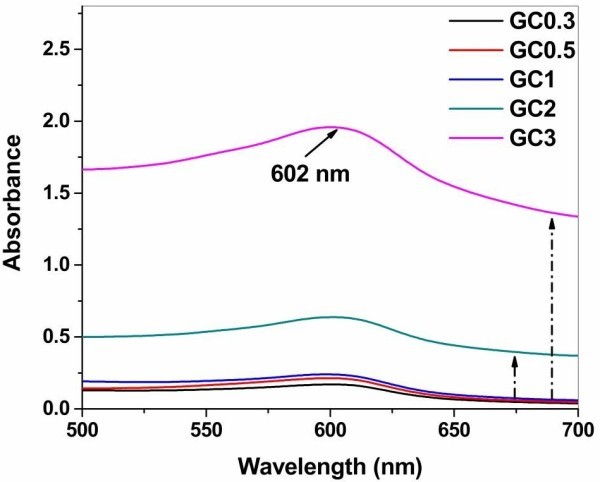
**UV absorption spectra of genipin dimer to show cross linkage formed in all composites GC0.3-GC3 (gelatin-chitosan in ratio 1:0.3 to 1:3).** The absorbance at 602 nm is marked with solid arrow line. The perpendicular dashed line indicates the shift from baseline for composites GC2 and GC3.

Generally, the water present in hydrogel is classified into two main types viz. free water and bound water. Free water can freeze at normal freezing point (0°C) with the heat enthalpy value 333.5 (J/g) (Kuo et al. 2005; Li et al. 2005). Meanwhile, the second type, bound water is subdivided into freezable (FB) and non-freezable (nFB) bound water molecules. Non-freezable bound water molecules involve neither both liquid–solid nor solid–liquid transition and in fact, is assumed to be integral part of polymers dispersed in hydrogel. The reason is that both of hydrogen atoms of water molecules are anchored with polymeric chain and the time of interaction is larger, in such a case, rather than free water (Feng et al. 2009; Loparo et al. 2006). The content of bound water increases depending on cross linkage and composition of polymeric components in blended hydrogel composites. In our work, native hydrogels were not cross linked using genipin whereas composites had well cross linked network by genipin dimer (as shown in Figure 1) and incorporation of copolymer, chitosan.

Genipin dimer formation was confirmed from the UV absorption peak at 602 nm. The composite GC3 shows maximum absorbance and is five times higher than the lower ratio of composites (GC0.3, GC0.5 and GC1) whereas the composite GC2 shows twice higher absorbance. Gelatin contains primary amino groups in amino acids such as lysine and arginine and participates in cross linkage with reactive amine groups of chitosan involving inter and intra cross linking up to particular percentage of copolymer blended. When chitosan percentage increases, they have the propensity to involve intra cross linkage within chitosan as availability of amino groups of gelatin is very less than that of chitosan. This leads to high level of cross linkage in polymeric network of hydrogel composites (GC2 and GC3).

Further, the free molecular motion in solution becomes less for GC2 and GC3 after 12 hours incubation. The mobility of polymeric components in these two composites is hindered significantly and that can be understood from the shift (indicated by perpendicular arrow) from base line observed in UV spectrum. This shows that genipin has strong impact in forming higher degree of cross linked network in composites GC2 and GC3 as the concentration of chitosan is increased. These cross linkage and blended polymers in composites have direct link with changes in dynamics of water present in hydrogels.

**Figure 2:**
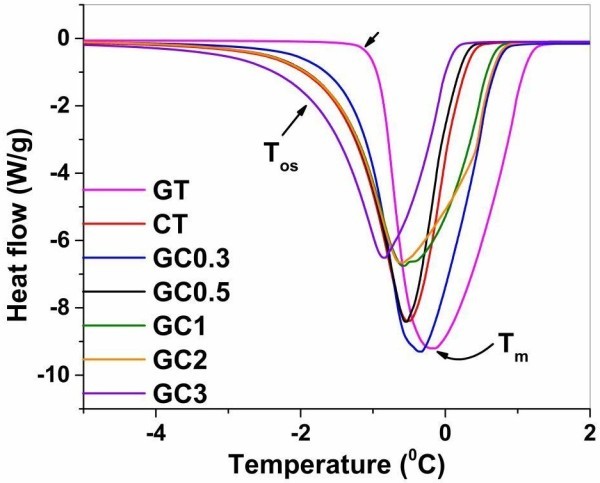
**DSC endothermic thermograms of water molecules in hydrogels with heat flow (W/g) and temperature (°C).** The linear arrow indicates onset temperature (T_os_) and curved arrow shows melting point (T_m_) of water confined into polymeric network of hydrogels (GT- gelatin; CT- chitosan; GC0.3-GC3 (gelatin-chitosan in ratio 1:0.3 to 1:3)).

Freezable bound water molecules play the vital role in lowering the temperature at which phase transition occurs because they share one hydrogen atom with polymer and another one with free water environment. Here, we have conducted DSC experiment for melting of solid water in hydrogels and endotherms for native and composites are shown in Figure 2. During crystallization, water molecules tend to align with hexagonal geometry, a most common and stable form (Gunko et al. 2013). As FB water molecules are partly attached with free water molecules, they however act as foreign solute significantly impeding the arrangement of geometrical network of crystallized ice. This is understood from the figure, in which onset temperature (represented as T_os_) of gelatin based hydrogels shifts to lower temperature when the ratio of chitosan is increased. It demonstrates that the larger the amount of FB water molecules, the greater the disruption in its geometrical alignment.

The lowering in melting point (T_m_) of water in hydrogels was also observed for each sample reflecting the onset temperature. The values for T_os_ and T_m_ are listed in Table 1. It is notable that native chitosan (CT) registered low value of onset and melting point when compared to GC0.3 and gelatin (GT). The reason is that 0.5% CT has more bound water molecules than 0.5% GT. In the case of composite GC0.3 (0.5% GT and 0.17% CT), the more hydrated gelatin dominates to yield less bound water content for each blended polymeric component containing chitosan. Indeed, the amount of bound water depends on the local environment generated in hydrogels. It is known that gelatin and chitosan alone too possess bound water and hence termed as hydrogels even in absence of cross linking agent and without blending with any copolymer. Chitosan increases bound water content for gelatin based hydrogels as NH_2_ and OH groups are located in each residue of polymer hence actively interact with water molecules. The percentage of freezable water content (free water and FB water) is calculated from the equation 1.
1

where, W_f_ and W_g_ are freezable water content and total weight (g) of hydrogels, respectively. ∆H is latent heat enthalpy of pure water and ∆H_m_ represents enthalpy of hydrogels.

**Table 1:** **The thermodynamic parameters and amount of freezable water of hydrogels determined from DSC**

Hydrogels ^*^	Onset temperature (°C)	Transition temperature	Enthalpy (J/g)	Amount of freezable water (%)
GT	−0.93	−0.17	319	95
CT	−1.39	−0.51	287	86
GC0.3	−1.22	−0.34	290	87
GC0.5	−1.43	−0.54	284	85
GC1	−1.51	−0.57	279	83.5
GC2	−1.53	−0.60	264	79
GC3	−1.79	−0.84	250	75

*(GT- gelatin; CT- chitosan; GC0.3-GC3 (gelatin-chitosan in ratio 1:0.3 to 1:3)).

As given in equations 2 and 3, the pore radii, R_p_ and their distribution in hydrogels through volume with respect to pore radii (dV/dR_p_) are evaluated from the thermodynamic values of water obtained, i.e. temperature (°C) and heat flow (W/g). It is assumed that the pores are spherical in colloidal hydrogel system having the condensate as water (Brun et al. 1977).
23

**Figure 3:**
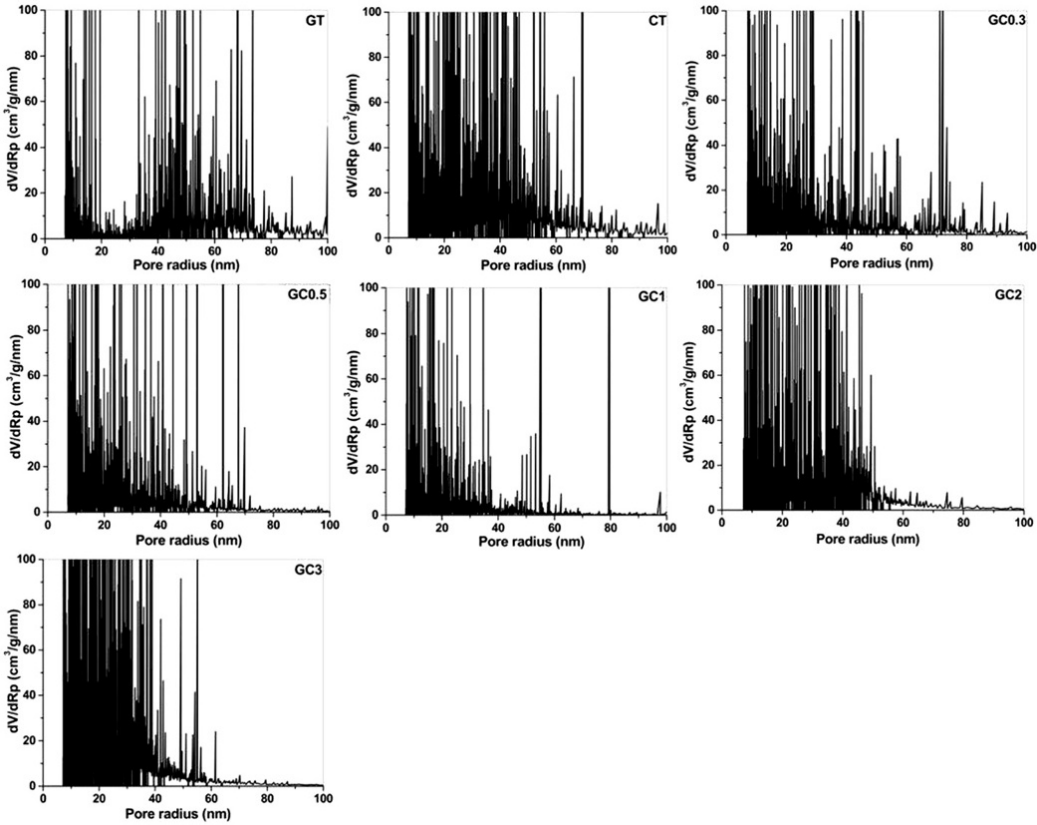
**Pore radii (R**_**p**_ **) and pore volume with respect to radii (dV/dR**_**p**_ **) distribution of various hydrogels.** (GT- gelatin; CT- chitosan; GC0.3-GC3 (gelatin-chitosan in ratio 1:0.3 to 1:3)).

**Figure 4:**
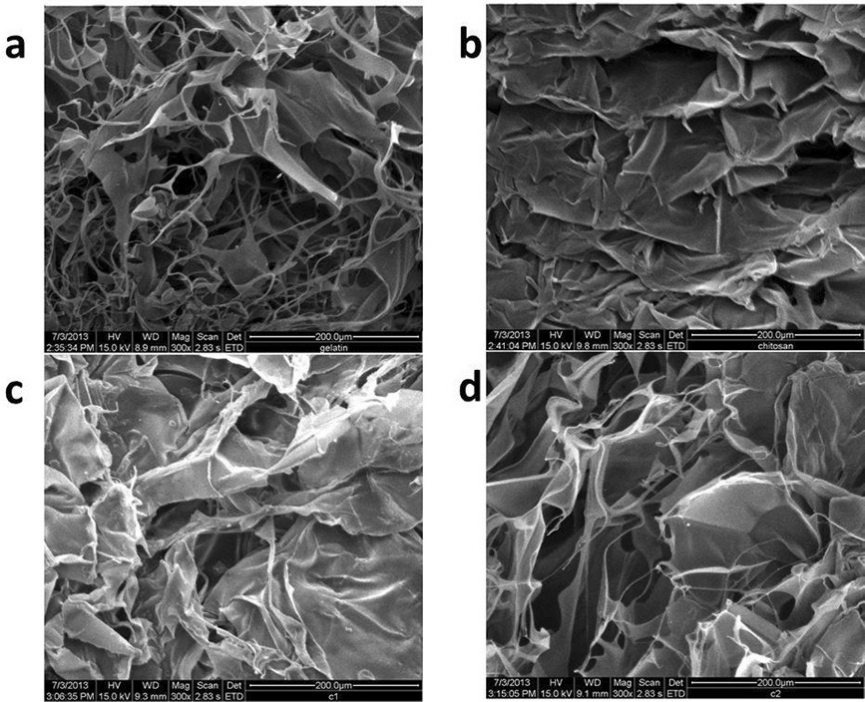
**SEM micrographs of lyophilized hydrogels.** Native hydrogels **a)** GT- gelatin **b)** CT- chitosan and **c)** GC0.3- (gelatin : chitosan, 1:0.3) and **d)** GC3- (gelatin : chitosan, 1:3).

Where, ∆T is the difference between temperature at any point (T_i_) and normal melting point (T_0_) of water. R_i_ and R_i+1_ are pore radii corresponding to temperature at successive points of temperature. ∆V is pore volume (cm^3^/g) at a temperature interval.As shown in Figure 3, the pore radii of nano sized pores in each hydrogel are measured. Though all the hydrogels are having pores in the range of 7 to 100 nm, GC2 and GC3 have pore radii in the range 7–50 and 7–40 nm, respectively, due to impact of genipin cross linkage with increasing concentration of chitosan. As can be seen in figure, the intensified peaks correspond to availability or number of pores with same radii. For GT, large numbers of pores are found within the range 40–55 nm. The gradual decrease in intensity of peaks is observed for CT indicating the numbers of pores with smaller radii are larger than that of higher radii. The pore size measured for CT, GC0.3, GC0.5 and GC1 were 7–100, 7–100, 7–80 and 7–60 nm, respectively. When chitosan is incorporated into gelatin, all the composites are showing more intensified peaks towards lower radii range. Hence, it is understood that chitosan has imparted more and reduced porous network for this gelatin based hydrogel system with the aid of genipin cross linkage. Though CT and GC0.3 are showing similar range of pore radii, the intensity of peaks of CT is more than GC0.3 at lower radii range reflecting the content of bound water measured through thermodynamic values.When thermoporometry technique was employed to manipulate nano sized pores, SEM studies were carried out to observe micro sized pores. As can be seen, in Figure 4, the pore radii of GT, CT, GC0.3 and GC3 were 2–15, 15–28, 15–25 and 5–20 μm, respectively. The micro sized pores seen in SEM images of lyophilized hydrogels are not corresponding to the values of nano sized pores and its variation, which is based on thermodynamics of water, depending on ratio of composites. The reason might be that the possibility of forming bound water molecules in micro sized pores is becoming less and hence it is not necessary the nano sized pores should be matched with micro sized pores of same hydrogels.

AC impedance measurement is helpful to know the nature of each hydrated polymeric components in hydrogel through its dielectric response under electric field. Here, we have given the comparative study of orientation polarization experienced by polymeric component of all hydrogels. This was studied from admittance (S), impedance (Ω) and permittivity (ϵ) functions which are dependent on each other by the following equations 4, 5, 6 and 7.
4567

Where, Z′(ω) and Z″(ω) are real and imaginary components of the total impedance, Z(ω), respectively. Y′(ω) is real part and Y″(ω) is imaginary part of total admittance, Y(ω). ω is angular frequency and equivalent to 2πf (Kanungo et al. 2013b; Manikoth et al. 2012). Where, ϵ_0_ is permittivity of free space (8.85 × 10^−12^ F/m) and relative permittivity is a unit less quantity. Capacitance of vacuum is denoted as C_0_ and *i* is an imaginary unit, (−1)^1/2^.

**Figure 5:**
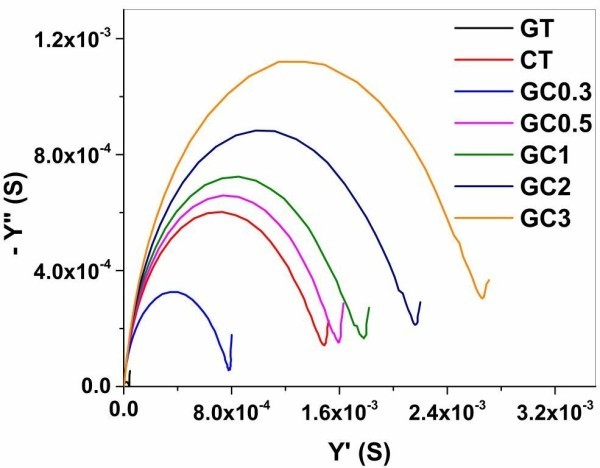
**Nyquist admittance plot drawn between real (Y′) and imaginary parts (−Y′′) of admittance function (S).** The steepness of circle indicates the charge binding capacity of polymeric network in hydrogels (GT- gelatin; CT- chitosan; GC0.3-GC3 (gelatin-chitosan in ratio 1:0.3 to 1:3)).

Admittance is described as accessibility of electric charge to polarize polymeric component in hydrogel. The water molecules cannot be polarized in the frequency range 10^−2^ – 10^5^ Hz, as they require higher frequency range (300 MHz – 300 GHz). Orientation polarization of polymeric component depends on its net dipole moment. The amphoteric nature of gelatin provides less dipole moment though they contain various charged, polar and non- polar amino acid residues. It varies depending on pH of solution above or below the isoelectric point. As isoelectric point of gelatin B, we have taken is 5.4 and hence GT prepared in doubled distilled water brings the pH around 7. The deprotonated state of gelatin imparts little net dipole moment. On contrary to this, Chitosan polymers contain a lot of reactive amine groups that lead to high net dipole moment having much tendency to be polarized. When chitosan is incorporated into gelatin, the dominance of chitosan is manipulated by increase in dipole moment of blended polymeric component in composites. These explanations are justified through Nyquist admittance plot and Debye relaxation model. Nyquist admittance plot demonstrates the capability of polymeric network in hydrogels to trap electric charge through steepness of circles whereas Debye relaxation model explains the polarization behavior of hydrated polymers as a response to electric charge entrapped. As shown in Figure 5, gelatin has low admittance value compared to chitosan. As the presence of gelatin overwhelms the concentration of chitosan, GC0.3 alone has the steep curve between GT and CT.

**Figure 6:**
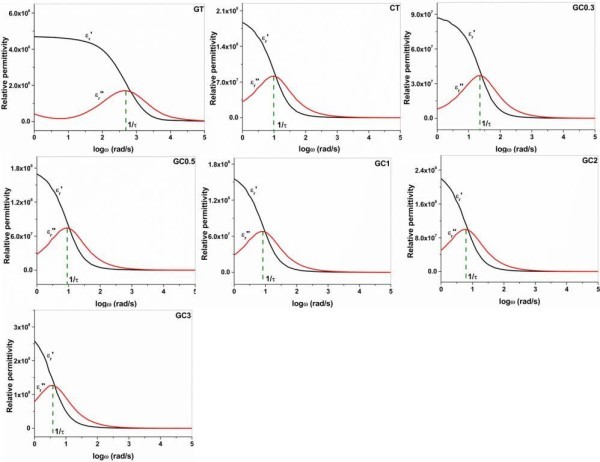
**Debye relaxation model with relative permittivity (** **ϵ**_**r**_ **) and logarithm of angular frequency (log** **ω).** The dotted line indicates the angular frequency point in which maximum relaxation time (τ) is encountered by hydrogels. The curves of real and imaginary relative permittivity are symbolized by ϵ_r_′ and ϵ_r_′′, respectively. (GT- gelatin; CT- chitosan; GC0.3-GC3 (gelatin-chitosan in ratio 1:0.3 to 1:3)).

As admittance is directly related to permittivity function as given in equation 6, the response of electric charged entrapped polymeric components polarize correspondingly. Debye relaxation model exemplified dielectric relaxation through permittivity function (Hougham et al. 1994; Wolf et al. 2012). Dielectric relaxation time (τ = 1/ ω) is the time taken by the polarizable entities as they tend to move to a new equilibrium state when applied field is removed or if frequency is altered. Though polymeric chain can migrate randomly without any external field, they tend to oscillate at particular frequency with the applied electric field. The lesser the dielectric relaxation time the larger the interaction of polymeric components with each other in hydrogel. As pointed out in Debye relaxation model (Figure 6), the maximum relaxation time exerted by the hydrogels is derived from the angular frequency, which meets the magnitude of imaginary relative permittivity (ϵ_r_″) curve that would face to middle portion of real relative permittivity (ϵ_r_′) curve. Dielectric relaxation values for GT, CT and composites are 0.002, 1.010, 0.730, 1.050, 1.090, 1.260 and 1.700 seconds, respectively. GT has very low relaxation time, which denotes much flexibility of protein hydrated chain in solution. Whereas, the relaxation time of CT is 500 times greater than that of GT. As the concentration of gelatin is high in GC0.3, the relaxation time is slightly reduced when compared to CT, whereas higher relaxation time is observed for GC3.

### Reflection of porosity and dielectric properties on *in vitro*drug delivery

The influence of dielectric properties and porosity on the drug delivery from hydrogel scaffolds was investigated by comparing with *in vitro* drug release profile. The percentage of drug released during washing was 40, 30, 34, 28, 27, 23 and 21% for GT, CT and composites in the order from GC0.3 to GC3, respectively. The less interactive drug molecules with polymeric components and drugs residing into micro sized pores are expected to come out during washing. As incorporation of chitosan and genipin cross linkage helps lowering pore size for the composites, they are retaining much amount of drugs into scaffold even after washing. The composite GC0.3, as expected, expels out higher amount of drugs through the scaffold than CT.

**Figure 7:**
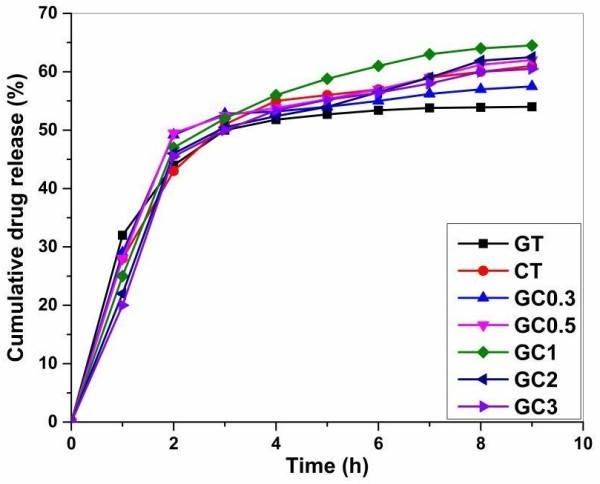
**The** ***in vitro*** **model drug release profile for native and composites during de-loading.** Cumulative drug release (%) estimated for 9 hours. (GT- gelatin; CT- chitosan; GC0.3-GC3 (gelatin-chitosan in ratio 1:0.3 to 1:3)).

The second part, de-loading describes how the drugs encapsulated into nano sized pores having strong interaction with polymeric components releases to the external medium. The initial release of drugs in the first hour reflects the order, as in washing part. They were 32, 28, 29, 28, 25, 22 and 20% for GT, CT and GC0.3-GC0.5, respectively (Figure 7). The cross linked networks in composites would help retain and release the drugs at sustained level leading to high amount of cumulated drugs released, at the end of experiment. In this particular 9 hours trial conducted, GC1 (64.5%) shows higher amount of release than other composites GC0.3 (57.5%), GC0.5 (62%) and native hydrogels (GT- 56.3%, CT- 61.2%). As expected, GC2 (62.5%) and GC3 (60.5%) did not eliminate entire amounts encapsulated. Their total release was lower than GC1. The reason might be that high degree of cross linkage (as shown in UV spectrum) releases very little amount of drugs at every hour and may last for longer duration with trace amounts. The results of *in vitro* model drug release are corresponding to genipin cross linkage, nano and micro sized pores, interaction and electric dipole moment of polymeric components in hydrogels.

## Conclusions

This study reveals that cross linking using genipin and increasing the concentration of chitosan leads to the reduction in pore size and increased orientation polarization of gelatin-chitosan polymeric components in hydrogels. Porosity has been explored through the presence of various types of water molecules in hydrogels using thermoporometry. Alternative current impedance analysis gave insights into dielectric properties and yielded information in terms of admittance and relaxation time. We have highlighted how different trends such as slow, immediate and sustained release of drugs from various hydrogels (*in vitro* drug delivery) correspond to porosity and dielectric properties studied. The results indicate that investigating porosity and dielectric properties would help to design hydrogels for intended drug delivery.

## Methods

Materials: Gelatin, Type B (bloom strength 125, pI 5.4) for Bacteriology, was purchased from Himedia. Genipin, chitosan, low molecular weight (75% deacetylated) and dialysis tubing cellulose membrane (1.3 inch, M.W cut-off 14,000) were obtained from Sigma Aldrich. Ethanol absolute (99.9%) and acetic acid used throughout the study were of analytical grade.

### Preparation of hydrogel composites and drug solution

Gelatin stock solution (3%) was prepared dissolving in warmth double distilled water at 40°C. Dissolution of chitosan was achieved in 2% acetic acid to have the same percentage of gelatin as stock solution. Genipin stock solution (0.1%) was made by dissolving in 40% ethanol. Native hydrogels, gelatin (GT) and chitosan (CT) were prepared separately without addition of cross linking agent to have final concentration 0.5% in water. Various gelatin-chitosan (GC) composites were prepared in such a way to have final concentration of gelatin as 0.5% with different percentage of chitosan. The ratio between gelatin and chitosan in composites was 1:0.3, 1:0.5, 1:1, 1:2 and 1:3 and were labelled as GC0.3, GC0.5, GC1, GC2 and GC3, respectively. Final concentration of genipin was maintained to be 0.01% in all the composites. During composite preparation, the polymer and co-polymer were stirred to get homogenized solution at first and genipin was added drop wise for one hour at 50°C, under constant stirring. The composites thus prepared were kept on stirring for 12 hours at 25°C for the completion of cross linkage. Dialysis was done against double distilled water to purify hydrogels. Catechin was dissolved in 20% ethanol in water to get the final concentration of 12 mM in solution. The tubes containing drug solution were covered instantly with dark brown sheet to be intact of light as much as possible.

### Thermoporometry measurement

Prior to thermoporometry measurement, the intensity of color formed in hydrogels was measured to know degree of cross linkage between polymers with the help of Shimadzu UV- Visible spectrophotometer UV-1800. The experiment was run in the range between 200 and 800 nm at 25°C. Thermoporometry measurement was carried out using Differential Scanning Calorimetry (DSC) by means of Q200 TA instrument. Initially, hermetically encapsulated aluminum pan was utilized for placing the samples and frozen to −25°C. Following this, endothermic thermogram was conducted with the temperature range from −25 to 5°C at a heating rate of 0.5°C/ min. The melting temperature (T_m_), onset temperature (T_os_) and total enthalpy (H_m_) for the phase transition of water in hydrogels were computed using the system generated software. All the experiments were done in triplicate and the mean was taken for calculation. From the thermograms, the pore radii were calculated as given in Results and discussion section below. Scanning Electron Microscopy (Hitachi S-3400 SEM microscope) was used for observation of micro sized pores. All the hydrogels were freeze dried and lyophilized previously to obtain hydrogels in dried form. The surface of the lyophilized samples was captured with the operating condition 15 kV and 300 X magnifications.

### AC impedance analysis

AC impedance analysis was carried out to evaluate dielectric properties of native and composites by means of CH instrumental electrochemical analyzer CHI-model 660D (U.S.A). The electrode set up consisted of glassy carbon electrode as working electrode, platinum electrode as counter electrode and saturated calomel electrode as the reference electrode. Open circuit potential (O.C.P) was measured for each sample, prior to AC impedance analysis and it was set as initial electric field (E) during the process. The other operating conditions were temperature (t) = 25°C, low frequency (Hz) = 10^−2^, high frequency (Hz) = 10^5^, amplitude (V) = 0.005, quite time (s) =2 and (0.1-1 Hz) = 1 Cycles; (0.01-0.1Hz) = 1 Cycles; (0.001-0.01 Hz) = 1 Cycles. All the experiments were done in triplicates and average values were reported.

### *In vitro*drug delivery

Drug solutions were added with all the hydrogels and incubated for 10 hours, at 60 rpm placing in an incubation shaker in dark room. Dialysis membrane was cut into equal length enough to hold the hydrogels. Pretreatment such as submerging the membrane in warmth EDTA solution at 80°C followed by rinsing with hot water was done for ensuring the membrane contains no trace amount of metals and glycerol to protect catechin during *in vitro* release. Furthermore, it creates well porous network in membrane for diffusion of drug molecules with ease. Washing the hydrogels was done against deionized water to remove the unbound drug molecules at 60 rpm at 25°C for one hour. Subsequently de-loading was also carried out against phosphate buffer saline (1X) raising the temperature to 37°C with 115 rpm. All the arrangements were made to mimic the physiological condition of human body. The amount of drug released (%) during washing and de-loading was calculated from the UV absorbance value around 279 nm.

## References

[CR1] Baba S, Osakabe N, Natsume M (2001). In vivo comparison of the bioavailability of (+)-catechin, (−)-epicatechin and their mixture in orally administered rats. J Nutr.

[CR2] Brun M, Lallemand A, Quinson JF (1977). A new method for the simultaneous determination of the size and the shape of pores: thermoporometry. Thermochim Acta.

[CR3] Chae E, Gim J, Song J (2013). Mesoporous manganese dioxide cathode prepared by an ambient temperature synthesis for Na-ion batteries. RSC Adv.

[CR4] Chaubaroux C, Vrana E, Debry C (2012). Collagen-Based Fibrillar Multilayer Films Cross-Linked by a Natural Agent. Biomacromolecules.

[CR5] Cheng Y, Nada AA, Valmikinathan CM (2014). In situ gelling polysaccharide-based hydrogel for cell and drug delivery in tissue engineering. J Appl Polym Sci.

[CR6] Cui L, Jia J, Guo Y (2014). Preparation and characterization of IPN hydrogels composed of chitosan and gelatin cross-linked by genipin. Carbohydr Polym.

[CR7] Dean DA, Ramanathan T, Machado D (2008). Electrical impedance spectroscopy study of biological tissues. J Electrost.

[CR8] Dongargaonkar AA, Bowlin GL, Yang H (2013). Electrospun Blends of Gelatin and Gelatin-Dendrimer Conjugates As a Wound-Dressing and Drug-Delivery Platform. Biomacromolecules.

[CR9] Fathima NN, Dhathathreyan A, Ramasami T (2002). Mercury Intrusion Porosimetry, Nitrogen Adsorption, and Scanning Electron Microscopy Analysis of Pores in Skin. Biomacromolecules.

[CR10] Feng J, Su W, Wang H-F (2009). Facile Fabrication of Diblock Methoxy Poly(ethylene glycol)-poly(tetramethylene carbonate) and Its Self-Assembled Micelles as Drug Carriers. ACS Appl Mater Interfaces.

[CR11] Gun’ko VM, Savina IN, Mikhalovsky SV (2013). Cryogels: Morphological, structural and adsorption characterisation. Adv Colloid Interface Sci.

[CR12] Hougham G, Tesoro G, Viehbeck A (1994). Polarization Effects of Fluorine on the Relative Permittivity in Polyimides. Macromolecules.

[CR13] Kanungo I, Chellappa N, Fathima NN (2011). Calorimetric analysis of gelatine-glycosaminoglycans blend system. Int J Biol Macromol.

[CR14] Kanungo I, Fathima NN, Rao JR (2013). Dielectric behavior of gelatine-glycosaminoglycans blends: An impedance analysis. Mater Sci Eng C.

[CR15] Kanungo I, Fathima NN, Rao JR (2013). Hydration dynamics of collagen/PVA composites: Thermoporometric and impedance analysis. Mater Chem Phys.

[CR16] Kuo J-L, Klein ML, Kuhs WF (2005). The effect of proton disorder on the structure of ice-Ih: A theoretical study. J Chem Phys.

[CR17] Landry MR (2005). Thermoporometry by differential scanning calorimetry: experimental considerations and applications. Thermochim Acta.

[CR18] Li W, Xue F, Cheng R (2005). States of water in partially swollen poly(vinyl alcohol) hydrogels. Polymer.

[CR19] Loparo JJ, Roberts ST, Tokmakoff A (2006). Multidimensional infrared spectroscopy of water. II. Hydrogen bond switching dynamics. J Chem Phys.

[CR20] Manikoth R, Kanungo I, Fathima NN (2012). Dielectric behavior and pore size distribution of collagen-guar gum composites: Effect of guar gum. Carbohydr Polym.

[CR21] Ngo D-H, Ryu B-M, Kim S-K (2014). Active peptides from skate (Okamejei kenojei) skin gelatin diminish angiotensin-I converting enzyme activity and intracellular free radical-mediated oxidation. Food Chem.

[CR22] Park M-R, Seo B-B, Song S-C (2013). Dual ionic interaction system based on polyelectrolyte complex and ionic, injectable, and thermosensitive hydrogel for sustained release of human growth hormone. Biomaterials.

[CR23] Parvez S, Rahman MM, Khan MA (2012). Preparation and characterization of artificial skin using chitosan and gelatin composites for potential biomedical application. Polym Bull (Heidelberg, Ger).

[CR24] Peng C-C, Burke MT, Chauhan A (2012). Transport of Topical Anesthetics in Vitamin E Loaded Silicone Hydrogel Contact Lenses. Langmuir.

[CR25] Pulieri E, Chiono V, Ciardelli G (2008). Chitosan/gelatin blends for biomedical applications. J Biomed Mater Res Part A.

[CR26] Qu F, Zhu G, Huang S (2006). Effective controlled release of captopril by silylation of mesoporous MCM-41. ChemPhysChem.

[CR27] Ren Y, Lam DCC (2008). Properties and Microstructures of Low-Temperature-Processable Ultralow-Dielectric Porous Polyimide Films. J Electron Mater.

[CR28] Rodrigues S, Dionisio M, Lopez CR (2012). Biocompatibility of chitosan carriers with application in drug delivery. J Funct Biomater.

[CR29] Sarkhejiya NA, Baldaniya LH (2012). Hydrogels: a versatile drug delivery carrier systems. Int J Pharm Sci Nanotechnol.

[CR30] Schaefer M, Gross W, Ackemann J (2002). The complex dielectric spectrum of heart tissue during ischemia. Bioelectrochemistry.

[CR31] Shi X-Y, Tan T-W (2004). New contact lens based on chitosan/gelatin composites. J Bioact Compat Polym.

[CR32] Soni H, Singhai AK (2013). Formulation and development of hydrogel based system for effective delivery of rutin. Int J Appl Pharm.

[CR33] Sun L, Wang Y, Jiang T (2013). Novel Chitosan-Functionalized Spherical Nanosilica Matrix As an Oral Sustained Drug Delivery System for Poorly Water-Soluble Drug Carvedilol. ACS Appl Mater Interfaces.

[CR34] Van Thienen TG, Raemdonck K, Demeester J (2007). Protein Release from Biodegradable Dextran Nanogels. Langmuir.

[CR35] Veluri R, Weir TL, Bais HP (2004). Phytotoxic and antimicrobial activities of catechin derivatives. J Agric Food Chem.

[CR36] Wang Q-S, Xiang Y, Cui Y-L (2012). Dietary blue pigments derived from genipin, attenuate inflammation by inhibiting LPS-induced iNOS and COX-2 expression via the NF-κB inactivation. PLoS ONE.

[CR37] Wolf M, Gulich R, Lunkenheimer P (2012). Relaxation dynamics of a protein solution investigated by dielectric spectroscopy. Biochim Biophys Acta Protein Proteonomics.

[CR38] Yamamoto T, Endo A, Inagi Y (2005). Evaluation of thermoporometry for characterization of mesoporous materials. J Colloid Interface Sci.

[CR39] Yamamoto T, Mukai SR, Nitta K (2005). Evaluation of porous structure of resorcinol-formaldehyde hydrogels by thermoporometry. Thermochim Acta.

[CR40] Yin G-B, Zhang Y-Z, Wang S-D (2010). Study of the electrospun PLA/silk fibroin-gelatin composite nanofibrous scaffold for tissue engineering. J Biomed Mater Res Part A.

[CR41] Zeugolis DI, Raghunath M (2010). The physiological relevance of wet versus dry differential scanning calorimetry for biomaterial evaluation: a technical note. Polym Int.

[CR42] Zhang Z, Chen X, Chen L (2013). Intracellular pH-Sensitive poly(ethylene glycol)-block-Acetalated-Dextrans as Efficient Drug Delivery Platforms. ACS Appl Mater Interfaces.

